# Genome Sequence of the Homoacetogenic, Gram-Negative, Endospore-Forming Bacterium *Sporomusa acidovorans* DSM 3132

**DOI:** 10.1128/genomeA.00981-17

**Published:** 2017-09-21

**Authors:** Jonathan R. Humphreys, Rolf Daniel, Anja Poehlein

**Affiliations:** a Clostridia Research Group, BBSRC/EPSRC Synthetic Biology Research Centre (SBRC), School of Life Sciences, University of Nottingham, Nottingham, United Kingdom; b Genomic and Applied Microbiology & Göttingen Genomics Laboratory, Georg-August University Göttingen, Göttingen, Germany

## Abstract

*Sporomusa acidovorans* DSM 3132 is a strictly anaerobic, spore-forming and acetogenic bacterium, which was isolated from effluent of an alcohol distillation fermenter. The genome harbors genes involved in the Wood-Ljungdahl pathway for carbon fixation and several genes for glycerol metabolism. The genome (6.06 Mb) contains 4,506 predicted protein-encoding genes.

## GENOME ANNOUNCEMENT

Within the phylum *Firmicutes* the class *Negativicutes* comprises the strictly anaerobic genus *Sporomusa*. Species of this genus are Gram-negative, endospore-forming bacteria, which are able to degrade a wide range of substrates, including the primary alcohols methanol and ethanol, as well as *N*-methyl compounds such as betaine and *N*,*N*-dimethylglycine (1). *Sporomusa* strains are of industrial interest due to their homoacetogenic metabolism using H_2_-CO_2_ as sole energy source to produce acetate via the Wood-Ljungdahl pathway (1, 2). *Sporomusa acidovorans* DSM 3132 was isolated from a pilot fermenter used in alcohol distillation (3). This strain is a potential candidate for industrial use as it is able to degrade glycerol (3), a by-product of the biodiesel industry (4). The genome sequences of acetogens contributed to the unravelling of the metabolism of these organisms, which in turn provides the basis for genetic engineering approaches (5–8). Here, we report the draft genome of *S. acidovorans* DSM 3132.

Chromosomal DNA of *S. acidovorans* DSM 3132 was isolated using the MasterPure complete DNA purification kit as recommended by the manufacturer (Epicentre, Madison, WI, USA). The extracted DNA was used to generate Illumina shotgun paired-end sequencing libraries, which were sequenced with a MiSeq instrument and the MiSeq reagent kit version 3, as recommended by the manufacturer (Illumina, San Diego, CA, USA). Quality filtering using Trimmomatic version 0.32 (9) resulted in 2,367,326 paired-end reads. The assembly was performed with the SPAdes genome assembler software version 3.5.0 (10). The assembly resulted in 176 contigs (>500 bp) and an average coverage of 79.03-fold. The assembly was validated and the read coverage determined with QualiMap version 2.1 (11). The draft genome of *S. acidovorans* DSM 3132 consisted of a single chromosome (6,060,615 bp) with an overall G+C content of 44.58%. Automatic gene prediction and identification of rRNA and tRNA genes were performed using the software tool Prokka (12). The draft genome contained 10 rRNA genes, 99 tRNA genes, 4,506 protein-encoding genes with predicted functions, and 1,263 genes coding for hypothetical proteins.

The gene cluster encoding the enzymes involved in the Wood-Ljungdahl pathway for carbon fixation were present in the genome of *S. acidovorans* DSM 3132 and showed the same orientation as the corresponding one of *S. ovata* H1 DSM 2662 (13). In contrast to *S. ovata* H1 DSM 2662, the genome of *S. acidovorans* DSM 3132 was larger and contained genes involved in glycerol metabolism. These included the enzymes glycerol kinase and glycerol-3-phosphate dehydrogenase, which convert glycerol to *sn*-glycerol 3-phosphase and subsequently to dihydroxyacetone phosphate (14), and genes encoding NAD-dependent glycerol 2-dehydrogenase, which converts glycerol to dihydroxyacetone (15), and glycerol dehydratase, which converts glycerol to 3-hydroxypropionaldehyde (16).

### Accession number(s).

This whole-genome shotgun project has been deposited at DDBJ/EMBL/GenBank under the accession no. LSLL00000000. The version described here is the first version, LSLL01000000.
